# Barriers to Geriatric Oral Health: A Multifaceted Public Health Issue

**DOI:** 10.7759/cureus.89604

**Published:** 2025-08-08

**Authors:** Athira Murali, Sapna C Muddappa, Rakesh R Rajan, Asha Joseph, Arjun B Ravi

**Affiliations:** 1 Department of Conservative Dentistry and Endodontics, Amrita Vishwa Vidyapeetham, Amrita School of Dentistry, Kochi, IND

**Keywords:** access to care, aging population, caregiver awareness, cognitive impairment, dementia, dental anxiety, dental care barriers, geriatric dentistry, multimorbidity, polypharmacy

## Abstract

Oral health is important for the overall health of an individual, particularly older adults. However, a number of obstacles frequently prevent older people from receiving timely and appropriate dental care. These obstacles are intricate and multifaceted, involving systemic diseases, cognitive elements, and psychological, financial, and educational issues. Dementia and Alzheimer's disease are examples of cognitive impairments that can make it difficult for an elderly person to seek or cooperate with dental treatment. Additional psychological factors that decrease care-seeking behaviour include anxiety, fear of dental treatments, depression, and a general lack of motivation. Financial constraints are major deterrents, such as low income and no dental insurance. Furthermore, polypharmacy and multimorbidity not only make treatment planning more difficult, but they also deprive oral health of priority. The problem is made worse by systemic healthcare barriers like inadequate referral systems, a lack of geriatric-focused dentists, and poor integration between dental and medical services. Neglect is also exacerbated by older adults' and their caregivers' perceived lack of need for dental care. A comprehensive, multidisciplinary strategy is needed to address these issues, one that incorporates training dental professionals in geriatric care, better public health regulations, caregiver education, and the creation of easily accessible, reasonably priced services. Improving the general and oral health of the elderly requires an understanding of and commitment to removing these obstacles.

## Introduction and background

Globally, the number of older people is increasing at an accelerated pace. Healthy aging has become a major concern for both individuals and society due to demographic changes in the Western world [1]. Between 2015 and 2050, the proportion of the world's population over 60 years of age will increase from 12% to 22% [2].

Oral problems affect over 3.5 billion people globally [3]. They become more prevalent as the number of elderly people increases. With age, there are changes in food preferences and nutritional habits due to medications and hyposalivation, impacting overall health and the quality of life, and can also lead to inadequate dental health, such as dental caries and periodontal disorders [4]. Furthermore, one out of four elderly people experiences a fall every year, which significantly increases their risk of dental trauma injuries from falls [5].

Oral health affects the general health and well-being of individuals, and it is, thus, receiving attention on a global scale [6]. Elderly people frequently overlook their dental health [7]. Identifying and managing dental health issues is essential, which is made practicable by routine dental exams. However, accessing adequate dental care is still a major challenge for many seniors, which results in notable variations in health outcomes [8]. Therefore, this review analyses the many barriers faced by the geriatric population in accessing oral healthcare.

## Review

Methods

Search Strategy

To develop this review, an extensive literature search was carried out to identify publications addressing obstacles in geriatric oral healthcare and strategies to overcome them. The search encompassed major databases such as PubMed/MEDLINE (Medical Literature Analysis and Retrieval System Online), Scopus, Cochrane Library, Google Scholar, and Web of Science. The search strategy used was: ("geriatric dentistry" OR "older adults" OR "elderly" OR "aging population")AND("oral health" OR "dental care" OR "oral hygiene" OR "oral health services")AND("barriers" OR "access to care" OR "dental anxiety" OR "cognitive impairment" OR "financial constraints" OR "healthcare integration")AND("public health" OR "oral health disparities" OR "oral care utilization").

Inclusion Criteria 

Articles written in English and published between 1990 and 2025 were included in the review. Reports, systematic reviews, narrative reviews, and original research addressing the barriers to oral health in older persons (≥60 years) were considered. Research concentrating on challenges linked to patients, finances, access to care, systemic healthcare, or education was given priority. Articles that offered methods or solutions to overcome the barriers in geriatric dentistry were chosen.

Exclusion Criteria

Research that focused solely on pediatric or non-geriatric adult populations (<60 years) was excluded. Unrelated or non-dental medical research that does not concentrate on oral health was not considered. Letters, editorials, or commentary that lack facts were excluded from the review.

Results

Patient-Related Barriers

Physical limitations: The process of developing and maintaining functional ability that enables well-being in older age is a crucial component of being self-sufficient [1,9,10]. The ability to move freely and carry out daily tasks, including standing, walking, and transferring, is referred to as mobility. These abilities are essential for preserving one's independence and standard of living. Mobility impairments are becoming more prevalent in elderly people, impacting over 35% of those over 70 and most people over 85 [11]. Furthermore, limited mobility might make it more difficult to get necessary services, such as medical and dental treatment, which contributes to inequality in health among the elderly population^[12]^. 

Neurological conditions: As people age, they may experience significant oral health issues because of diminished motor skills. Parkinson's disease (PD) is a neurodegenerative disorder in which motor function deteriorates. Neuronal loss in particular brain regions is one neuropathological characteristic of PD[13]. When contrasted with healthy people, patients with PD have lower oral hygiene, periodontal health, and caries prevalence [14]. They experience xerostomia, tremors, stiffness, bradykinesia (slowness of movement), and postural instability, which are some of the typical signs, and all of these can significantly impair a person's capacity to carry out everyday duties, including maintaining good dental hygiene. They end up facing difficulty in performing oral health due to poor dexterity [15].

Cognitive and psychological factors: Dementia is responsible for more than 50% of cases diagnosed in individuals over the age of 65. The neurological condition known as Alzheimer's disease (AD) worsens with time [16]. More than 50 million people globally have been reported to have AD [17]. According to Rejnefelt et al. [18] and Delwel et al. [19], patients with dementia often have oral health issues. Elevated levels of cognitive impairment appear to be linked to declined oral health, which is characterized by increased production of plaque and increasingly prevalent oral diseases [20]. As dementia progresses, it often brings more than just memory loss. Many elderly patients begin to show behavioral and psychological symptoms like agitation, restlessness, and even resistance to simple daily routines like brushing their teeth. For caregivers and dental professionals, these changes can make routine oral care extremely difficult [21]. In some cases, these individuals may be suffering from dental pain or oral infections but are unable to express it. A toothache might go unnoticed. Gums may bleed, but the patient cannot explain what they feel. Over time, these hidden issues begin to affect how they eat, how they speak, and even how they feel. Something as simple as a dry mouth or a sore tooth can drastically reduce their quality of life; however, it may never be reported or treated [22].

Dental anxiety or fear: Dental anxiety leads to a vicious circle of dental avoidance, whereby those who experience dental anxiety miss more appointments, visit the dentist less frequently, and are more likely to self-medicate. A study showed that over two-thirds of older persons only went to the dentist when they had an urgent oral health issue [23]. This might be because they have to depend on others for transport, and also because of lower income and elevated price of dental visits (just 10.3% of survey participants had health insurance, and 63.1% of them said they did not have enough money). Another study examined the reasons for dental anxiety and the fear of going to the dentist, and the most often cited causes were discomfort during dental treatments, shame about tooth form, fear of infection, and fear of bleeding[24].

Depression or lack of motivation to seek care: According to the WHO, major depressive disorder is expected to be the leading cause of illness burden globally by 2030[25]. Depression is the third most prevalent cause of disability globally and the most prevalent mental disorder affecting older persons [26,27]. In a comprehensive evaluation of 26 publications, Kisely et al. reported that those with anxiety and depression are about two times as probable as the general population to suffer from serious dental conditions, such as periodontal disease and dental caries. They are also more likely to have decayed, missed, or filled teeth, primarily as a result of irregular dental appointments and poor oral hygiene habits [28].

Multimorbidity and polypharmacy: Multimorbidity, according to the WHO, is two or more chronic health conditions in the same individual [29]. Because of the age distribution of the population, multimorbidity affects a larger proportion of those under 65 years of age in various communities. Broader socioeconomic issues also influence the occurrence; individuals from poor backgrounds are more likely to suffer multimorbidity early than their more wealthy peers [30]. Older persons sometimes use five or more medications at the same time, a condition known as polypharmacy [31]. Elderly people with polypharmacy often notice a decline in salivary flow. According to a review, the frequency of dental cavities is increased among the elderly by 60% due to low resting pH and low stimulated salivary flow rate[32].

Lack of perceived need: According to a study by Manski et al., individuals who were more focused on maintaining their oral health visited the dentist regularly [33]. Reduced utilization of dental and oral health services appears to be a result of the low emphasis placed on them and also the ignorance concerning the elderly's demand for dental and oral care [34]. According to Mittal et al., misconceptions such as oral health not being part of physical health and people without teeth not needing to see a dentist result in a decline in oral health and dental care utilization [35]. Such beliefs are deeply ingrained, often passed down through generations, and are rarely challenged unless education and awareness are actively promoted. Many elderly individuals may accept oral discomfort, poor denture fit, or gum bleeding as a normal part of aging, when in fact, these conditions are preventable and manageable with timely professional care. The result is a cycle of neglect. Low perceived need leads to low service utilization, which in turn leads to worsening oral health outcomes [36].

Financial Barriers

The expense of receiving dental and oral care is one of the deterrents. Studies by Soares et al. [37], Meneses-Gómez et al. [38], and Ferreira et al. [39] found that the percentage of persons who utilized oral and dental treatments rose in tandem with income. Dental care puts households under additional financial strain and may make it impossible for them to pay for it or be motivated to use it[37].

For many older adults, the situation becomes even more challenging due to their limited or non-existent post-retirement income, which is usually prioritized for basic needs such as housing, food, and medications. Hence, older adults frequently utilized fewer dental and oral services [40]. The financial strain of dental care on elderly households can lead to difficult decisions such as delaying or foregoing treatment entirely.

Access-to-Care Barriers

Transportation: Transportation was the largest indirect expense for elderly people. Those without relatives or friends to drive them to appointments or accompany them had particular difficulties with this. In a study, non-attendance was frequently seen in the case of elder participants because of the inability to get someone to accompany them to the dentist [41]. Many elderly individuals may feel unsafe, anxious, or disoriented when traveling alone, particularly when public transportation is unreliable, inaccessible, or unfamiliar. This further discourages attendance, particularly for preventive care, which may not seem urgent in the absence of pain or visible symptoms[42].

Geographic barriers: Compared to urban dwellers, rural inhabitants have less likelihood of having previous preventative dental treatments, according to 2016 Medical Expenditure Panel Survey (MEPS) data [43]. Rural populations may lose out on timely dental care, which might negatively impact their oral health. Access to preventive dental care is considerably more restricted for racial/ethnic minority groups residing in more remote areas. Also, a shortage of dental personnel and the need for transport to a dental hospital could be other barriers.

Limited availability of geriatric-focused dentists: A scoping review on geriatric dental health by Brandt et al. evaluated the postgraduate knowledge-seeking patterns in dentists and the role of undergraduate programs in influencing attitudes and showed varied results [44]. In their review, some studies found that younger dentists were more motivated and ready to serve elderly and medically compromised patients [45], while other studies found that younger dentists had a negative outlook toward elderly patients and were less inclined to treat elderly patients who were in poor physical condition [46]. Willingness to treat influences how a physician will ultimately handle a senior dental patient. This encompasses knowledge of aging, attitude, and empathy towards elderly patients and the perception of skill in caring for the elderly[47].

Systemic Healthcare Barriers

The accelerated aging of current populations leads to a rise in demand for social and health care [48-50]. Having sufficient access to extended care, community service, and healthcare is crucial for geriatric adults to age healthily [51]. Current care systems focus greater on treating particular diseases than on offering collaborative person-centered care; they are not sufficiently matched to the complicated needs of the elderly, including those with chronic ailments [52,53]. The inadequate integration between the dental and medical care systems is one of the main systemic issues in geriatric dentistry. Dental services are still mostly isolated from general healthcare, which leads to fragmented care for older adults with numerous co-morbidities, even though oral-systemic health connections are becoming more widely acknowledged [54,55]. Elderly patients need the care of a team of specialists to manage their variety of chronic illnesses, and their care may also involve third-sector carers and informal carers, which inevitably results in fragmented care. Frequently, this treatment is poorly coordinated, with each doctor focusing on their own specialty without giving enough thought to the patient's overall treatment plan and health. In the end, this causes subpar care, inferior patient outcomes, and unnecessarily high health expenditures due to multiple medications, drug interactions, conflicting medical advice, and needless repetition of diagnostic procedures or treatments [55,56].

Educational Barriers

Modern healthcare is built on patient education and involvement, particularly among older patients. Health results and general quality of life can be significantly improved by teaching patients about their conditions, course of treatment, potential adverse effects, and the need to adhere to treatment [57]. Patients who receive more information are frequently more involved, asking thoughtful questions and discussing symptoms or worries clearly. Additionally, they are better equipped to make educated judgments, which helps them steer clear of any issues that can result from miscommunications regarding their treatments or health state [58]. The term "gerontechnology" has been used to characterize a collection of technology intended to help older people age in place, maintain their independence, and account for age-related declines and limitations [59]. A wide range of technologies is included in digital health, such as wearable technology, mHealth, telemedicine, and remote monitoring systems [60].

**Figure 1:**
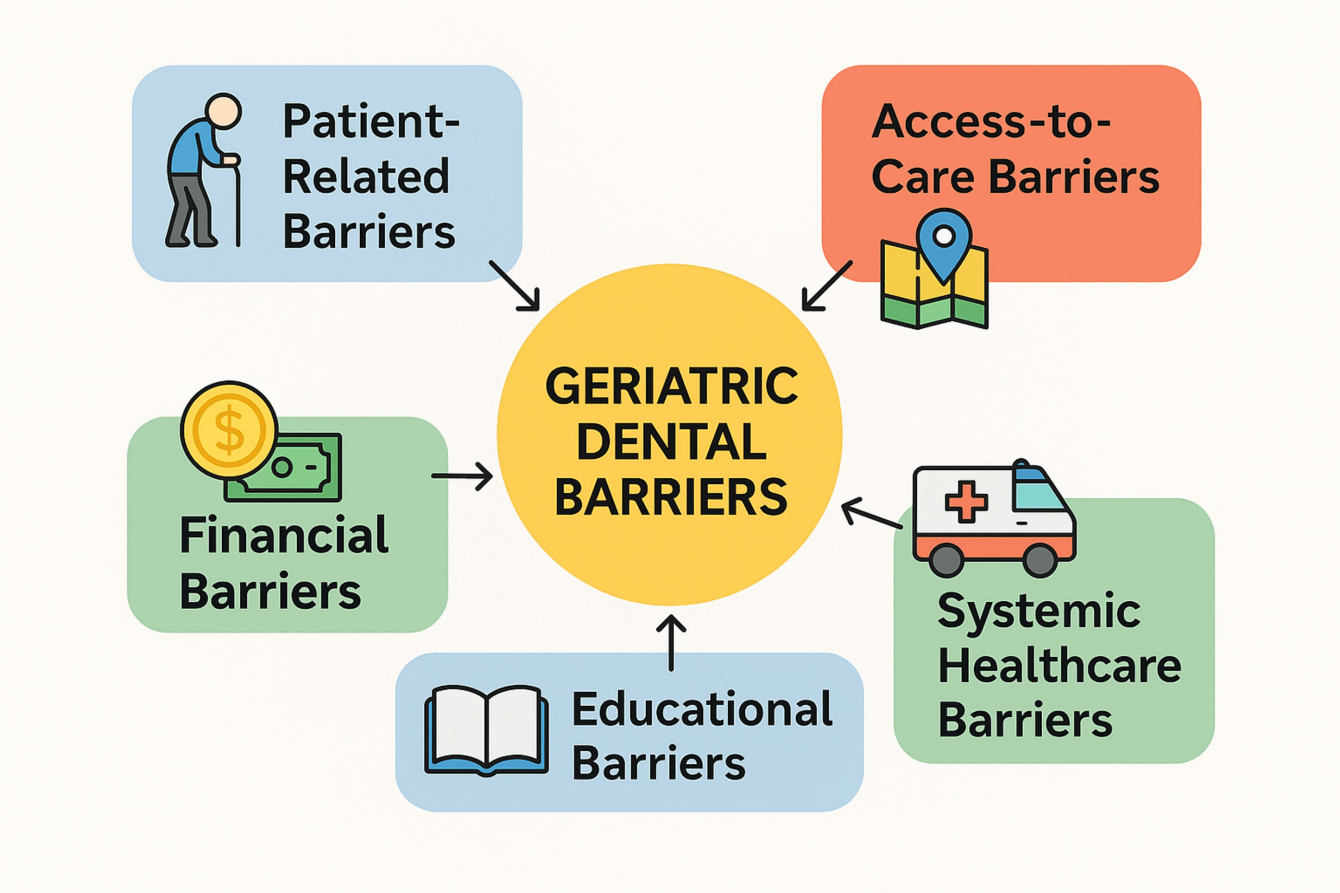
Diagrammatic representation of various geriatric dental barriers Image Credit: Authors; created using Canva (www.canva.com)

The various barriers to geriatric oral health are given in Table 1 and Figure 1.

**Table 1:** Summary of barriers to geriatric oral health

Category	Description	Studies
Physical limitations	Age-related decline in mobility, reduced access to dental care and independence.	Ferrucci et al., 2016 [9]; Brabrand et al., 2018 [10]
Neurological Barriers (PD & Dementia)	Diminished motor skills and poor dexterity, declined oral health, and difficulty in expressing pain	Kalia and Lang, 2015 [13]; Verhoeff et al., 2023 [14]; Rejnefelt et al., 2006 [18]
Dental Anxiety & Fear	Dental fear and anxiety to seek treatment, self-medication, irregular appointments, and poor oral hygiene habits	Hassan et al., 2022 [24]; Kisely et al. 2016 [28]
Financial & Systemic Barriers	Limited income, lack of insurance, rural access challenges, and fragmented healthcare, reduced dental service utilisation.	Soares et al. 2021 [37]; Luo et al. 2021 [43]
Access-to-Care Barriers	Transportation issues, geographic disparities, a shortage of geriatric-focused dentists, and limited availability of timely and specialized care.	Borreani et al., 2008 [41]; Kiyak and Reichmuth, 2005 [42]; Luo et al., 2021 [43]; Brandt et al., 2025 [44]; Fiske et al., 1990 [45]; Major et al., 2016 [46]
Educational & Awareness Barriers	Poor health literacy and misconceptions, reduced perceived need for care.	Bhattad and Pacifico, 2022 [57]; Bouma, 1998 [59]
Overcoming Barriers	Compassionate care, geriatric-focused training for dentists, financial assistance programs, mobile/community dental services	Borreani et al., 2008 [41]; Sharifi, 2024 [61]

Overcoming Barriers

Calm and soothing atmosphere: Various approaches can be adopted to mitigate these barriers effectively, which will help ensure a better quality of care for the elderly. The primary step that we can adopt is to create a comfortable and supportive clinical environment, which plays a significant role in geriatric dentistry. Dentists should maintain a warm and inviting atmosphere. Spend time calming the patient and explaining the course of treatment clearly, as older adults often appreciate a slower, more empathetic approach that will build their trust and ease their psychological distress. Understanding and acknowledging concerns related to previous dental experiences, along with reducing waiting time, contributes to a more positive perception of dentistry among the elderly [61,62]. 

Cognitive-friendly care: For patients with cognitive impairments, using visual aids such as diagrams and simple charts, along with brief, direct language, enhances understanding. Important information should be repeated and confirmed to ensure clarity and minimize confusion. Use of oral hygiene tools (e.g., electric toothbrushes with modified handles), involvement of caregivers, short and flexible appointment scheduling, and frequent professional cleanings can significantly improve their oral health. Collaboration between dentists, neurologists, caregivers, and speech-language therapists is also vital in ensuring a smooth treatment [40].

Patient-dentist rapport: Reduced waiting time translates into reduced anxiety. Measures like providing earplugs or quiet drills can help alleviate stress associated with dental procedures, such as the anxiety caused by drill noise. Building trust and rapport is essential. Older adults highly value courtesy and kindness, and often appreciate when treatments are not rushed. Spending time to converse and listen to their concerns enhances the treatment experience. Ensuring that patients are actively involved in selecting suitable treatment options empowers them and promotes better compliance [63,64].

Improving financial access: To promote better oral health among the elderly, it is essential to introduce initiatives such as free dental examinations and increased financial assistance for senior citizens. In several instances, elderly individuals choose not to see a dentist again to spare themselves the humiliation of not having enough money to pay for treatment. Hence, lowering the cost of dental care will improve access to treatment for the elderly and encourage regular dental visits. Due to a lack of knowledge about dental care costs, elderly patients frequently avoid dental visits out of concern for the expense. Hence, providing clear communication of fees to both existing and potential patients can help reduce the financial barriers many older adults face. Additionally, assisting with appointment scheduling and offering outreach services through dentists visiting community centres can further reduce the indirect costs and improve accessibility to care [61].

Enhancing physical and professional access: Accessibility is another key component. It is critical to guarantee physical access to dental treatment for elderly patients, including provisions for those with disabilities. In a study by Borreani et al., participants suggested that a mobile dental team visit day centres and community organisations to provide older people with dental examinations and transport them to the dental hospital if additional treatments were required in order to address the problems related to isolation and travel to dental clinics [41]. At the same time, there is a need to train dental professionals in geriatric care and improve public health regulations and caregiver education, ensuring a more holistic approach to elderly oral health.

Promoting awareness and compassionate care: Finally, raising awareness through the media is vital. Articles on geriatric dentistry should be published in newspapers and journals targeting older audiences. Educational presentations at local organizations and distribution of informative bulletins can demonstrate readiness and capability to treat medically compromised elderly patients. However, even with all of this help, patients still need to be motivated to communicate their requirements, control their fears, show up, and finish their treatment. Above all, elderly individuals require more than just clinical expertise; they need a caregiver who is both a competent professional and a compassionate friend. Blending professional care with a human touch remains one of the most effective approaches to treating this vulnerable population [65,66].

## Conclusions

As a result of the world's fast-aging population, there is an unparalleled rise of older people with complicated unmet health and social demands. This report has highlighted five major categories of barriers: patient-related, financial, access-to-care, systemic healthcare, and educational barriers, each playing a substantial role in limiting the effectiveness and reach of geriatric dental services.

A comprehensive and coordinated strategy, including healthcare professionals, caregivers, and community organizations, is needed to address these complex issues. To remove these barriers, it is imperative to improve provider training in geriatric care, increase insurance coverage, integrate oral health into general healthcare, and support community-based educational programs. By putting such strategies into practice, we may increase accessibility, lessen inequalities, and improve the general oral health and standard of living for the aging population.
